# Former ONTPD chairs perspective on the 2-year fellowship proposal

**DOI:** 10.1038/s41372-026-02805-8

**Published:** 2026-07-09

**Authors:** Rita M. Ryan, Josef Neu, Judy Aschner, Christiane E. L. Dammann, Patricia R. Chess, Kristina Reber

**Affiliations:** 1 Case Western Reserve University, Cleveland, OH USA; 2 University of Florida-Gainesville, Gainesville, FL USA; 3 Hackensack Meridian School of Medicine, Hackensack, NJ USA; 4 Tufts University, Medford, OR USA; 5 University of Rochester, Rochester, NY USA; 6 Baylor College of Medicine, Houston, TX USA

**Keywords:** Scientific community, Health occupations

## Abstract

Several former Organization of Neonatal-Perinatal Medicine Training Program Directors describe their concerns about the newly proposed 2-year fellowship in neonatology.

Dear Neonatal Perinatal Medicine community:

We are writing this commentary based on our individual long-standing commitment to the discipline of academic neonatology. The optimal structure and duration of Neonatal–Perinatal Medicine (NPM) fellowship training remain central to ongoing discussions about workforce sustainability, trainee well-being, and the future of academic neonatology. Recent proposals advocating for a 2-year, clinically focused fellowship pathway—sometimes coupled with a shortened pediatric residency—have emerged in response to concerns regarding training length and workforce shortages. While these concerns are valid, the implications of such changes extend beyond streamlining. We contend that maintaining a 3-year NPM fellowship is essential to preserve clinical excellence, ensure procedural competence, protect trainee well-being, and sustain the pipeline of physician-scientists who drive innovation and the future of neonatal critical care.

Neonatology is inherently complex, requiring both advanced cognitive skills and technical proficiency. Clinical decision-making in the neonatal intensive care unit (NICU) evolves through repeated exposure to diverse and often rare conditions, combined with longitudinal mentorship and graduated autonomy. A 3-year fellowship provides the necessary temporal framework for this progression, allowing trainees to move beyond algorithmic management toward nuanced, experience-informed judgment. In contrast, a compressed 2-year model risks prioritizing service demands over reflective learning and clinical synthesis. *Clinical expertise in high-acuity environments cannot be accelerated without compromising depth and the safety of patients*.

This concern is further compounded by recent changes in pediatric residency training. Contemporary pediatric residents frequently have limited exposure to the NICU, and evolving duty structures—including the removal of NICU night call requirements in some programs—have reduced opportunities for hands-on procedural experience and gradual transition into a less supervised environment. As a result, incoming fellows may begin subspecialty training with less familiarity with neonatal physiology and fewer procedural skills than prior cohorts. In this context, shortening fellowship training is not a compensatory adjustment but an additional constraint. Core competencies such as airway management, chest tube placement, and vascular access require repetition and deliberate practice over time. A 2-year fellowship, even with increased clinical density, is unlikely to provide sufficient opportunities for mastery. Consider a typical fellow managing a critically ill preterm infant requiring emergent airway stabilization and central access. Competence in such scenarios is not achieved through isolated exposure but through iterative experience across varied clinical contexts. The additional year of training allows fellows to refine both technical execution and situational judgment—skills that are essential for independent practice but difficult to compress into a shorter timeframe.

Beyond clinical training, the most significant impact of a shortened fellowship may be on scholarly development. The third year of NPM fellowship has traditionally provided additional protected time for research and other scholarly activities, including quality improvement, medical education, point-of-care ultrasound (POCUS) training, involvement in advocacy, and advanced evidence appraisal. This period is foundational rather than supplementary. It allows fellows to develop critical thinking skills, engage in hypothesis-driven inquiry, and build the academic competencies necessary for careers in research, education, advocacy, and leadership.

Importantly, many neonatologists who become successful investigators do not enter fellowship with a defined research trajectory. For many, meaningful engagement with research begins during fellowship, often catalyzed by the required scholarly activity and protected scholarly time afforded by a 3-year program. Eliminating or substantially reducing this period risks narrowing the pool of future physician-scientists, particularly those who lacked prior research exposure during medical school or residency. Historical and contemporary examples within neonatology demonstrate that fellowship can serve as a pivotal entry point into academic/research careers (e.g., (by their own report) Richard Martin and Satyan Lakshminrusimha, among many others).

The long-term implications for the field are substantial. Neonatology has benefited from sustained contributions by clinician-investigators who have advanced understanding in areas such as respiratory support, neonatal nutrition, microbiome science, and neurodevelopmental outcomes. A shift toward predominantly clinical training risks diminishing this tradition. Reduced research exposure during training is associated with lower rates of academic career pursuit, decreased grant funding success, and diminished scholarly output. While a 2-year model may modestly increase the number of practicing clinicians in the short term, *it may simultaneously erode the academic foundation that underpins evidence-based neonatal critical care*.

Concerns about trainee well-being and recruitment further challenge the feasibility of a 2-year fellowship. Compressing approximately 18 months of clinical service into a 2-year period—effectively 22 months when accounting for leave—creates an intensive training environment that may be perceived as unsustainable. Prospective applicants increasingly prioritize work–life balance and may be deterred by a model that intensifies clinical demands without providing adequate time for recovery, family issues, or professional development. Paradoxically, this pathway, designed to address workforce shortages, could reduce the overall interest in the specialty.

An alternative approach, modeled after training structures in other subspecialties such as child neurology, involves shortening pediatric residency to two years while maintaining a 3-year NPM fellowship. This “2 + 3” model preserves the integrity of subspecialty training while reducing total training duration and allowing earlier transition to attending-level compensation. It also avoids excessive compression of clinical service and retains dedicated time for scholarly activity.

Such a model offers additional advantages. Portions of pediatric residency training, while essential for general pediatric competence, may be less directly aligned with the needs of future neonatologists. Rotations in some outpatient settings, though valuable, may not substantially contribute to Neonatology-specific expertise. A streamlined residency could allow earlier and more focused exposure to neonatal critical care, potentially improving both preparedness for fellowship and recruitment into the field. Admittedly, the “2 + 3” pathway may initially appeal to a limited subset of trainees, particularly given the relatively low exposure to neonatology during medical school. However, increased awareness of a shorter, more efficient and targeted training pathway could enhance interest over time. Efforts to expand early exposure to neonatal critical care and research will be critical to realizing this potential.

It is also important to recognize that workforce dynamics vary across pediatric subspecialties. While some disciplines face acute shortages, this is not universally the case. Broad implementation of shortened training pathways risks applying uniform solutions to heterogeneous challenges. In neonatology, where clinical complexity and academic productivity are deeply intertwined, preserving comprehensive training is particularly important.

Several actionable steps may help address current concerns without compromising training quality. Medical schools and pediatric departments should expand early exposure to pediatrics and neonatology, including opportunities for clinical engagement and research. Residency programs should seek to enhance meaningful NICU experiences, ensuring that trainees enter fellowship with a stronger clinical foundation. Finally, national organizations and training leaders should carefully evaluate the long-term consequences of altering fellowship duration, particularly with respect to research capacity and academic workforce sustainability.

In light of these considerations, proposals for a 2-year, clinically focused NPM fellowship should be approached with caution. While well-intentioned, such models are unlikely to provide a durable solution to workforce challenges and may introduce unintended consequences that compromise both training quality and the future of the field.

In conclusion, the 3-year NPM fellowship remains essential for developing skilled, adaptable, and academically engaged neonatologists. It provides the clinical depth, procedural expertise, and scholarly foundation necessary to meet the evolving demands of neonatal critical care. Alternative pathways that preserve these core elements—such as the “2 + 3” model—deserve thoughtful exploration. However, a transition to a 2-year fellowship risks undermining both the immediate and long-term goals of the speciality. Maintaining the 3-year structure represents a deliberate investment in the continued excellence and advancement of neonatal–perinatal medicine.

Respectfully,

Former ONTPD Chairs:

Rita M. Ryan, MD, Professor of Pediatrics, Case Western Reserve University

Josef Neu, MD, Professor of Pediatrics, University of Florida-Gainesville

Judy Aschner, MD, Professor of Pediatrics, Hackensack Meridian School of Medicine

Christiane E.L. Dammann, MD, Professor of Pediatrics, Tufts University

Patricia R. Chess, MD, MS, HPE, Professor of Pediatrics, University of Rochester

Kristina Reber, MD, Professor of Pediatrics, Baylor College of Medicine

